# Supplementary material for: Integrated Analysis of the Lung Microbiome and Metabolome Reveals Associations Between Amino Acid Metabolism and Pulmonary Fibrosis in a Bleomycin-Induced Mouse Model
Source: Int J Mol Sci. 2026 Jun 30;27(13):5895. doi: 10.3390/ijms27135895 (PMC13362081; doi:10.3390/ijms27135895)
Supplement: Supplementary file 1 [file ijms-27-05895-s001.zip › result/4.MetDiffAnalysis/Kmeans_diff/all/Diff_kmeans_all_subcluster/kmeans_subcluster_plots.pdf]

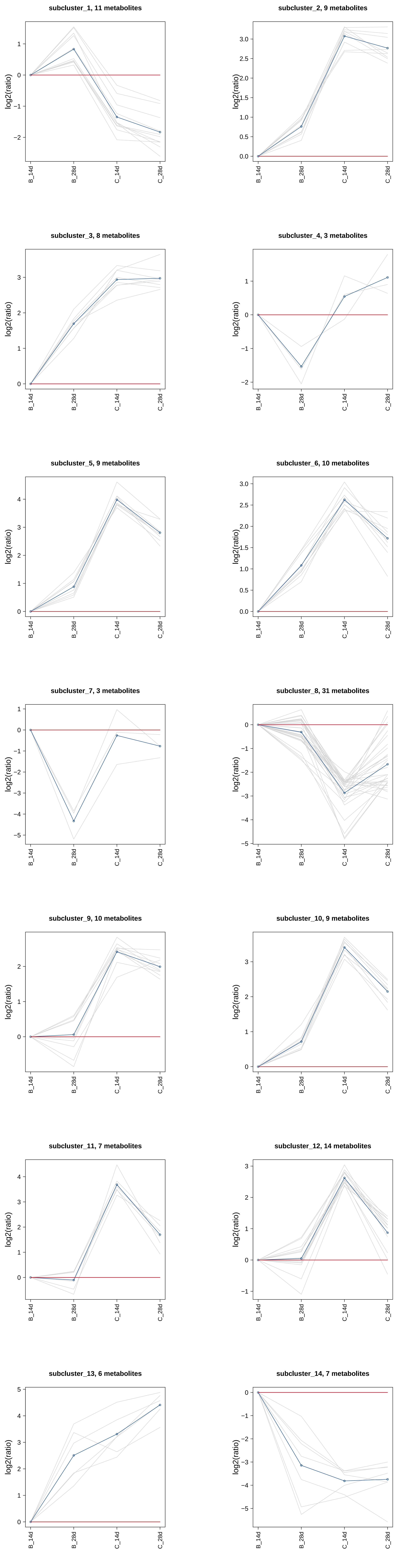

subcluster_1, 11 metabolites
subcluster_2, 9 metabolites
subcluster_3, 8 metabolites
subcluster_4, 3 metabolites
subcluster_5, 9 metabolites
subcluster_6, 10 metabolites
subcluster_7, 3 metabolites
subcluster_8, 31 metabolites
subcluster_9, 10 metabolites
subcluster_10, 9 metabolites
subcluster_11, 7 metabolites
subcluster_12, 14 metabolites
subcluster_13, 6 metabolites
subcluster_14, 7 metabolites
log2(ratio)
B_14d
B_28d
C_14d
C_28d
